# Human coronavirus alone or in co-infection with rhinovirus C is a risk factor for severe respiratory disease and admission to the pediatric intensive care unit: A one-year study in Southeast Brazil

**DOI:** 10.1371/journal.pone.0217744

**Published:** 2019-06-03

**Authors:** Alessandra K. Matsuno, Talita B. Gagliardi, Flavia E. Paula, Luciano K. S. Luna, Bruna L. S. Jesus, Renato T. Stein, Davi C. Aragon, Ana P. C. P. Carlotti, Eurico Arruda

**Affiliations:** 1 Department of Pediatrics, Ribeirão Preto Medical School, University of São Paulo, Ribeirão Preto, Brazil; 2 Department of Cell Biology and Virology Research Center, Ribeirão Preto Medical School, University of Sao Paulo, Ribeirão Preto, Brazil; 3 Department of Pediatrics,Pontifícia Universidade Católica do Rio Grande do Sul School of Medicine, Porto Alegre, Brazil; University of North Carolina at Chapel Hill, UNITED STATES

## Abstract

**Objective:**

We aimed to assess the profile of respiratory viruses in young children hospitalized for acute lower respiratory tract infection (ALRI) and its association with disease severity, defined as need for pediatric intensive care unit (PICU) admission.

**Design:**

Prospective observational cohort study.

**Setting:**

A tertiary-care university hospital in Brazil.

**Patients:**

Children younger than three years attending the pediatric emergency room with ALRI who were admitted to the hospital.

**Interventions:**

None.

**Measurements and main results:**

Nasopharyngeal aspirates were collected from patients from June 1^st^, 2008 to May 31^st^, 2009within the first 48 hours of hospitalization. Nasopharyngeal aspirates were tested for 17humanrespiratory viruses by molecular and immunofluorescence based assays. Simple and multiple log-binomial regression models were constructed to assess associations of virus type with a need for PICU admission. Age, prematurity, the presence of an underlying disease and congenital heart disease were covariates. Nasopharyngeal aspirates were positive for at least one virus in 236 patients. Rhinoviruses were detected in 85.6% of samples, with a preponderance of rhinovirus C (RV-C) (61.9%). Respiratory syncytial virus was detected in 59.8% and human coronavirus (HCoV) in 11% of the samples. Co-detections of two to five viruses were found in 78% of the patients. The detection of HCoV alone (adjusted relative risk (RR) 2.18; 95% CI 1.15–4.15) or in co-infection with RV-C (adjusted RR 2.37; 95% CI 1.23–4.58) was independently associated with PICU admission.

**Conclusions:**

The detection of HCoV alone or in co-infection with RV-C was independently associated with PICU admission in young children hospitalized for ALRI.

## Introduction

Acute lower respiratory tract infections (ALRI) are responsible for the death of approximately 160,000 neonates and over 760,000 infants annually [1].Respiratory syncytial virus (RSV) and rhinovirus (RV) are the most frequent causes of acute respiratory infections in children [2–4].Primary RSV infections may lead to severe bronchiolitis and pneumonia [5]. RV usually causes rhinopharyngitis[6], but may also cause illness of the lower respiratory tract and asthma exacerbations, and has been associated with severe ALRI in young children [7,8]. A few studies have investigated the viral profile in children attending the pediatric emergency room (ER) with ALRI and its association with disease severity [9–12], but finding vary. Moreover, the role of viral co-infections in illness severity has been controversial [10, 12, 13–15]. In young children who presented to an outpatient clinic or ER in Japan with acute respiratory illness, RSV, RV, parainfluenza viruses (PIV) and human metapneumovirus (HMPV) were the most prevalent viruses. Similarly to what was found in a previous study in Brazil [4], detection of RSV, alone or in co-detections, was associated with increased disease severity in children in Japan [9]. In Seattle, USA, RV was the most frequent virus detected in children less than three years old presenting to the ER with ALRI, and 52% of them required hospital admission [10]. In addition, RV viral load and co-detection with RSV was associated with more severe disease in that study [10]. In children younger than two years old who presented to a pediatric ER with ALRI in Malaysia, RSV and RV were the most frequently detected viruses and, although RSV was associated with a history of wheezing, virus detection was not associated with need for hospitalization [11]. In The Netherlands, the most frequent viruses detected in children presenting to the ER or outpatient clinic with ALRI were RSV, RV and human coronavirus (HCoV), and RSV detection correlated with longer duration of oxygen therapy [12]. However, there was no association between the virus species and hospital length of stay, and virus co-detections were not associated with disease outcome [12].In the present study, a comprehensive polymerase-chain-reaction(PCR) panel of primers and probes was used to detect respiratory viruses, enabling virus identification to the species level, in respiratory samples collected from children younger than three years with ALRI seen at the pediatric ER and subsequently admitted to hospital. Association of different respiratory viruses with disease severity, defined as need for admission to the pediatric intensive care unit (PICU), was assessed.

## Material and methods

### Patients and samples

This was a prospective cohort study conducted in a tertiary-care university hospital in Brazil. The study was approved by the Research Ethics Committee of the Clinical Hospital, School of Medicine of Ribeirão Preto, University of São Paulo, a tertiary-care facility in the city of Ribeirão Preto, state of Sao Paulo, Brazil. A written informed consent was obtained from parents/guardians (protocol number HCRP A07-020). All consecutive children younger than three years attending the pediatric emergency room with ALRI who were admitted to hospital from June 1^st^, 2008 to May 31^st^, 2009were eligible for the study. ALRI was defined by the presence of cough, tachypnea, respiratory distress with prolonged expiratory time, and wheezing or crackles on auscultation. Patients with a diagnosis of bacterial pneumonia as indicated by clinical presentation and chest X-ray findings or a positive blood culture were excluded from analysis.

Demographic, clinical and outcome data were collected from patients’ health records. Need for PICU admission was considered the main indicator of disease severity. Nasopharyngeal aspirates were collected from patients within the first 48 hours of hospitalization, as previously described [16] and they were split into aliquots: two 250μL aliquots mixed with 750μL of TRIzol (Invitrogen, Thermo Fisher Scientific, MA, USA) to DNA/RNA extraction and two 500μL backup aliquots mixed 1:1 with 500μL of viral transport medium, which consists of minimal essential medium with Eagle’s salts plus 20% fetal bovine serum, 15% glycerol and 1% antibiotic-antimycotic solution (GIBco, Thermo). All backup aliquots were stored at -70°C until analysis.

### Rapid screening for RSV

Nasopharyngeal aspirates were routinely screened for RSV, by eithera rapid chromatographic immunoassay for RSV antigen (Directigen EZ RSV Test, Becton Dickinson and Company, Franklin Lakes, NJ, USA) or indirect immunofluorescence (IF) assay. Directigen EZ RSV was performed on 250μL of nasopharyngeal aspirates following the manufacturer’s protocol. Indirect IF assay was performed with RSV-specific monoclonal antibody (MAb 858–4; Millipore, MA, USA) diluted 1:100 in phosphate-buffered saline (GIBco, Thermo), revealed with Alexa Fluor 488-labeled donkey anti-mouse IgG (Life Technologies, Carlsbad, CA, USA) diluted 1:200 in phosphate-buffered saline. Slide preparation and IF protocols are available at http://dx.doi.org/10.17504/protocols.io.w8rfhv6 and http://dx.doi.org/10.17504/protocols.io.w8ufhww.

### Detection of RNA and DNA respiratory viruses by real-time PCR

TRIzol aliquots from each sample were used to do DNA and RNA extractions. Total RNA was extracted following manufacturer’s protocol with some adaptations(http://dx.doi.org/10.17504/protocols.io.w8vfhw6), and DNA-enriched fractions were used for DNA purification using the Wizard Genomic DNA purification kit (Promega, Madison, WI, USA) as per manufacturer’s protocol. One microgram of total RNA was used in a reverse transcription reaction carried out with “Multiscribe reverse transcriptase” (Applied Biosystems, Thermo), primed with random hexamers and following manufacturer’s protocol. This random cDNA was used in TaqMan real-time PCR assays to detect RSV-A and B; HMPV A and B; human influenza viruses (FLU) A and B; and PIV 1 and 3. Human beta-actin housekeeping gene was used as internal controls in all assays. All PCR assays were performed using only one set of primers-probe per reaction, except for RSV and HMPV detection, which were tested in duplex format. Human bocavirus (HBoV) and human adenovirus (HAdV) were detected by qPCR in a single format assay (http://dx.doi.org/10.17504/protocols.io.w8yfhxw). HCoV detection was performed by a nested RT-PCR method that uses primers targeting the RNA-dependent RNA polymerasegene [17]. Rhinovirus (RV) detection wasperformed by a two-step PCR method (http://dx.doi.org/10.17504/protocols.io.w83fhyn) [18, 19].

All PCR reactions were performed on a Thermocycler 7300 (Applied Biosystems, Thermo), using published primers and probes sequences [20]. All real-time PCR plates included appropriate negative controls matched to every step of the testing of nasopharyngeal aspirates. Negative controls were total RNA or DNA extracted from uninfected Hela cells and ultrapure water treated in the same way as clinical samples.

### Statistical analysis

The analysis was made using SAS 9.4 (SAS/STAT User’s Guide, Version 9.4, Cary, NC: SAS Institute Inc., 2013). Data were expressed as median (range) or number (%). Patients were grouped according to the need for PICU admission. Continuous variables between groups were compared by Mann-Whitney U test and categorical variables, by Fisher’s exact test. Simple and multiple log-binomial regression models were constructed to assess associations of virus type with need for PICU admission. Relative risks (RR) and 95% confidence intervals (95%CI) were obtained after adjusting log-binomial regression models. Initially, simple log-binomial regression models were fitted, resulting in crude relative risks. Subsequently, the adjustment of multiple log-binomial regression models considering age, prematurity, the presence of an underlying disease and congenital heart disease as covariates, resulted in adjusted relative risks [21]. A 5% significance level was considered in all analysis.

## Results

Over the study period, nasopharyngeal aspirates were collected from 279 children seen at the pediatric emergency room with ALRI. Thestudy population comprised 236 patients who had at least one respiratory virus detected in respiratory specimens. All patients were hospitalized. Twenty-five percent of patients (n = 60) had comorbidities. The most common underlying diseases were neurological impairment (n = 17), congenital anomalies (n = 13) and chronic lung disease (n = 11). Forty-seven patients (19.9%) were admitted to the PICU; 25 of them (53.2%) received invasive mechanical ventilation for a median time of 7 days (range 1 to 99 days). Length of PICU stay ranged from 1 to 254 days (median 9.5 days). Demographic data were not significantly different between patients admitted to the PICU compared with those who did not need PICU admission. However, use of systemic antibiotics and the presence of underlying diseases and congenital heart disease were more frequent in patients admitted to the PICU, and they also had a longer hospital length of stay, Table 1.

**Table 1:** Demographic and clinical data.

Characteristic	All (n = 236)	PICU admission (n = 47)	No PICU admission (n = 189)
Age (months)	5.2 (0.2–35)	3.6 (0.2–35)	5.9 (0.3–34)
Age < 6 months	125 (53)	29 (61.7)	96 (50.8)
Weight (kg)	6 (2–21)	5.2 (2.1–14)	6.2 (2–21)
Male gender	89 (38)	18 (38.3)	71 (37.6)
Prematurity	43 (18.2)	7 (14.9)	36 (19)
Underlying disease	60 (25)	21 (44.7)	39 (20.6)*
Congenital heart disease	23 (9.7)	9 (19)	14 (7.4)*
Use of systemic antibiotics	153 (64.8)	37 (78.7)	116 (61.4)*
Length of hospital stay (days)	8 (1–254)	18 (3–254)	7 (1–210)*

Data are expressed as median (range) or n (%). PICU, pediatric intensive care unit.

*P < 0.05 for comparison between PICU admission and No PICU admission groups

The most frequently detected virus was RV (85.6%), followed by RSV (59.8%), HBoV (23.7%), HMPV (17.8%), HCoV (11.4%), HAdV (10.6%), PIV (10.2%) and FLU (8.5%). Co-detections were found in 182 (78%) patients, S1 Table.

The results of multiple log-binomial regression analyses showed thatthe detection of HCoV alone (adjusted relative risk (RR) 2.18; 95% CI 1.15–4.15) or in co-infection with RV-C (adjusted RR 2.37; 95% CI 1.23–4.58) was independently associated with PICU admission, S2 Table.

Eight patients (3.4%) died. Their median age was 3.5 months (range 2.3–9.3 months); six (75%) were female. Six patients (75%) who died had comorbidities:hydrocephalus (n = 3),Pompe disease (n = 1), Down syndrome (n = 1) and pulmonary hypoplasia (n = 1). Three patients had a single virus type detected in their respiratory samples (RV-C in two patients, and RV-A in one patient), two patients had dual viral co-detection (RV-C + RSV-B and RV-C + HCoV OC43), and three patients had triple viral co-detection (RSV-A + FLU-A + HBoV; RV-C + RSV-A + HMPV-A; and RV-C + HMPV-A + FLU-B). The causes of death were respiratory insufficiency (n = 4), cardiogenic shock (n = 2) and septic shock (n = 2).Of note, the patient who had the longest hospital stay (254 days) and also the longest duration of mechanical ventilation (99 days) had pulmonary hypoplasia, a severe underlying condition, and he ultimately died of sepsis.

## Discussion

In this study, viruses were detected in 85% of young children attending the emergency room with ALRI and subsequently admitted to the hospital. RV, especially RV-C, was the most frequently detected virus, followed by RSV. Twenty percent of patients were admitted to the PICU. Comorbidities and congenital heart disease were more frequent in patients who were admitted to the PICU. In addition, the detection of HCoV alone or in co-infection with RV-C was independently associated with PICU admission.

Similarly to our data, RV was also the most commonly detected virus in children younger than 3 years presenting to the Seattle Children’s Hospital pediatric ER with a symptomatic respiratory tract infection, and the majority of them had lower respiratory tract infection and required hospitalization [10]. RV was also the most prevalent virus detected in children aged two weeks to 5 years admitted to a hospital with ALRI in Germany, exceeding the frequency of RSV [22]. Moreover, RV-C has been reported as the most frequent RV species and has been associated with severe disease in children less than 3 years old [8, 22].

Viral co-detection was found in approximately three-quarters of our study population, with RV/RSV as the most frequent duo. However, we observed that viral co-detection per se was not a risk factor for PICU admission. In keeping with this, a recent systematic review and meta-analysis showed that respiratory viral coinfection was not associated with need for hospitalization, intensive care admission or length of stay in children [15]. In addition, the number of detected viruses has not been associated with illness severity [14]. Indeed, surprisingly, an inverse correlation between the clinical severity score and the number of viruses detected has been observed [23]. Nevertheless, a previous study performed in Southeast Brazil showed that coinfections, especially involving RSV, were associated with increased severity [13]. In the present study, RSV in co-detection with other viruses was not associated with increased disease severity.

HCoV was detected in 11% of children admitted to hospital with ALRI in our study, with a predominance of OC43 (40.7%) and 229E (33%)types. Although human coronaviruses most frequently cause common colds, they may cause severe respiratory diseases, such as severe acute respiratory syndrome [24]. We observed that one-third of children with HCoV infectionand 41% of patients withHCoV in co-infection withRV-C were admitted to the PICU. Furthermore, we found that the detection of HCoV alone or in co-infection with RV-C was an independent risk factor for PICU admission.Similar to our results, HCoV was detected in 8.2% of hospitalized children aged 3.2 ± 3.9 years with respiratory tract infection in New York, OC43 was the most prevalent type (40.1%), and 11% of patients with HCoVinfectionneeded PICU admission. Additionally, the presence of chronic complex underlying conditions, including cardiovascular, genetic and respiratory diseases was associated with increased disease severity [25], which is corroborated by our data.

Almost two-thirds of our children were treated with systemic antibiotics. This high frequency of antibiotic use is similar to that reported in developed countries [26, 27]. Empiric antimicrobial therapy, driven by unfavorable clinical conditions in the face of probable bacterial infections, is likely to be initiated in the absence of immediate laboratory confirmation of virus detection. In the present study, the only rapid diagnostic test for respiratory virus availableearly at hospital admission was the RSV rapid antigen detection test. Therefore, the development of clinically relevant rapid tests for respiratory viruses is needed and shall help antimicrobialstewardship programs.

Since the present study was done in a single health center, this raises concern that epidemiologic data may not be generalizable. While this limitation is acknowledged, the results obtained are similar to those reported from Europe and North America [8, 22].Another concern could be the assumption of disease etiology based on virus detection atone-time point. However, this is a practical diagnostic approach worldwide and all patients enrolled in the study had signs and symptoms of ALRI requiring hospitalization concomitantly with virus detection. It should also be noted that, although the diagnosis of bacterial pneumonia, as indicated by clinical presentation, chest X-ray findings or positive blood culture, was an exclusion criterion, it is difficult to discriminate viral and bacterial pneumonia in cases with negative bacterial cultures, because clinical presentation and chest radiography findings may overlap.

In conclusion, the detection of HCoV alone or in co-infection with RV-C was independently associated with PICU admission in young children hospitalized for ALRI.Rapid and reliable diagnostic tests for respiratory viruses associated with disease severity should be widely available to improve patient management and optimize healthcare resources.

## Supporting information

S1 Table Viruses detected in respiratory specimens of all patients (n = 236). (DOCX)

S2 Table Viruses detected in respiratory samples and risk for admission to the pediatric intensive care unit (PICU). (DOCX)
