# Supplementary material for: Human coronavirus alone or in co-infection with rhinovirus C is a risk factor for severe respiratory disease and admission to the pediatric intensive care unit: A one-year study in Southeast Brazil
Source: PLoS One. 2019 Jun 3;14(6):e0217744. doi: 10.1371/journal.pone.0217744 (PMC6546210; doi:10.1371/journal.pone.0217744)
Supplement: S1 Table — (DOCX) [file pone.0217744.s001.docx]

**Table 2:** Viruses detected in respiratory specimensof all patients (n=236)

| **Viruses** | **n (%)** |
| --- | --- |
| **Rhinovirus (RV)**  *RV-A*  *RV-B*  *RV-C* | 202 (85.6)  *52 (22)*  *4 (1.7)*  *146 (61.9)* |
| **Respiratory syncytial virus (RSV)**  *RSV-A*  *RSV-B*  *RSV-A and RSV-B*  *RSV unknown* | 141 (59.8)  *45 (19.1)*  *45 (19.1)*  *3 (1.3)*  *48 (20.3)* |
| **Human bocavirus** | 56 (23.7) |
| **Human metapneumovirus (HMPV)**  *HMPV-A*  *HMPV-B*  *HMPV-A and HMPV-B* | 42 (17.8)  *13 (5.5)*  *18 (7.6)*  *11 (4.7)* |
| **Human coronavirus (HCoV)**  *HCoV 229E*  *HCoV OC43*  *HCoV HKU*  *HCoV NL63* | 27 (11.4)  *9 (3.8)*  *11 (4.7)*  *5 (2.1)*  *2 (0.8)* |
| **Human adenovirus** | 25 (10.6) |
| **Human parainfluenza virus (PIV)**  *PIV-1*  *PIV-3* | 24 (10.2)  *18 (7.6)*  *6 (2.5)* |
| **Influenza virus (FLU)**  *FLU-A*  *FLU-B* | 20 (8.5)  *11 (4.7)*  *9 (3.8)* |
| **Co-detections**  *Dual co-detections*  *RV + RSV*  *Triple co-detections*  *Four viruses*  *Five viruses* | 182 (78)  *98 (53.3)*  *59 (61.2)*  *55 (29.9)*  *23 (12.5)*  *6 (3.3)* |
