# Supplementary material for: Human coronavirus alone or in co-infection with rhinovirus C is a risk factor for severe respiratory disease and admission to the pediatric intensive care unit: A one-year study in Southeast Brazil
Source: PLoS One. 2019 Jun 3;14(6):e0217744. doi: 10.1371/journal.pone.0217744 (PMC6546210; doi:10.1371/journal.pone.0217744)
Supplement: S2 Table — (DOCX) [file pone.0217744.s002.docx]

**Table 3:**Viruses detected in respiratory samples and risk for admission to the pediatric intensive care unit (PICU)

| **Virus type** | **PICU admission** | **No PICU admission** | **Crude RR (95% CI)** | **Adjusted RR (95% CI)** |
| --- | --- | --- | --- | --- |
| Rhinovirus  Negative  Positive | 5(14.7)  42(20.8) | 29 (85.3)  160 (79.2) | Ref.  1.41 (0.60-3.31) | Ref.  1.33 (0.58-3.05) |
| Rhinovirus-A  Negative  Positive | 41 (22.3)  6 (11.5) | 143 (77.7)  46 (88.5) | Ref.  0.52 (0.23-1.15) | Ref.  0.55 (0.25-1.22) |
| Rhinovirus-C  Negative  Positive | 12 (13.3)  35 (24) | 78 (86.7)  111 (76) | Ref.  1.80 (0.99-3.27) | Ref.  1.61 (0.89-2.90) |
| Respiratory syncytial virus  Negative  Positive | 21 (22.1)  26 (18.4) | 74 (77.9)  115 (81.6) | Ref.  0.83 (0.50-1.39) | Ref.  0.83 (0.50-1.37) |
| Human bocavirus  Negative  Positive | 37 (20.6)  10 (17.9) | 143 (79.4)  46 (82.1) | Ref.  0.87 (0.46-1.63) | Ref.  0.94 (0.51-1.75) |
| Human metapneumovirus  Negative  Positive | 40 (20.6)  7 (16.7) | 154 (79.4)  35 (83.3) | Ref.  0.80 (0.38-1.68) | Ref.  0.75 (0.37-1.52) |
| Human coronavirus  Negative  Positive | 38 (18.2)  9 (33.3) | 171 (81.8)  18 (66.7) | Ref.  1.83 (1.01-3.36) | Ref.  2.18 (1.15-4.15) |
| Parainfluenza virus  Negative  Positive | 44 (20.7)  3 (12.5) | 168 (79.3)  21 (87.5) | Ref.  0.60 (0.20-1.79) | Ref.  0.73 (0.24-2.21) |
| Human adenovirus  Negative  Positive | 45 (21.3)  2 (8) | 166 (78.7)  23 (92) | Ref.  0.37 (0.09-1.45) | Ref.  0.40 (0.10-1.58) |
| Influenza virus  Negative  Positive | 43 (19.9)  4 (20) | 173 (80.1)  16 (80) | Ref.  1.00 (0.40-2.51) | Ref.  1.14 (0.44-2.90) |
| Influenza virus-A  Negative  Positive | 45 (20)  2 (18.2) | 180 (80)  9 (81.8) | Ref.  0.91 (0.25-3.27) | Ref.  1.17 (0.32-4.23) |
| Influenza virus-B  Negative  Positive | 45 (19.8)  2 (22.2) | 182 (80.2)  7 (77.8) | Ref.  1.12 (0.32-3.91) | Ref.  1.08 (0.30-3.96) |
| Co-detection  No  Yes | 13 (24.1)  34 (18.7) | 41 (75.9)  148 (81.3) | Ref.  0.77 (0.42-1.36) | Ref.  0.77 (0.45-1.31) |
| Rhinovirus and respiratory syncytial virus  Negative  Positive | 23 (19.5)  24 (20.3) | 95 (80.5)  94 (79.7) | Ref.  1.04 (0.62-1.74) | Ref.  1.00 (0.61-1.64) |
| Rhinovirus-C and respiratory syncytial virus  Negative  Positive | 28 (19.9)  19 (20) | 113 (80.1)  76 (80) | Ref.  1.01 (0.60-1.70) | Ref.  0.94 (0.57-1.55) |
| Rhinovirus-C and human bocavirus  Negative  Positive | 43 (20.8)  4 (13.8) | 164 (79.2)  25 (86.2) | Ref.  0.66 (0.26-1.71) | Ref.  0.65 (0.25-1.68) |
| Rhinovirus-C and human metapneumovirus  Negative  Positive | 40 (19.5)  7 (22.6) | 165 (80.5)  24 (77.4) | Ref.  1.15 (0.57-2.35) | Ref.  1.11 (0.55-2.20) |
| Rhinovirus-C and human coronavirus  Negative  Positive | 40 (18.3)  7 (41.2) | 179 (81.7)  10 (58.8) | Ref.  2.25 (1.20-4.24) | Ref.  2.37 (1.23-4.58) |

Data are expressed as n (%).RR, relative risk;Ref., reference group.Adjusted relative risk was obtained by fitting a model considering age, prematurity, underlying disease and congenital heart disease as covariates.
